# An Innovative Harmonic Radar to Track Flying Insects: the Case of *Vespa velutina*

**DOI:** 10.1038/s41598-019-48511-8

**Published:** 2019-08-19

**Authors:** Riccardo Maggiora, Maurice Saccani, Daniele Milanesio, Marco Porporato

**Affiliations:** 1 Dipartimento di Elettronica e Telecomunicazioni, Politecnico di Torino, Torino, Italy; 2 Dipartimento di Scienze Agrarie, Forestali e Alimentari, Università degli Studi di Torino, Torino, Italy

**Keywords:** Ecosystem ecology, Invasive species

## Abstract

Over the last 30 years, harmonic radars have been effective only in tracking insects flying at low altitude and over flat terrain. We developed an innovative harmonic radar, implementing the most advanced radar techniques, which covers a large field of view in elevation (with an angular aperture of about 24°) and can track insects up to a range of 500 m. We show all the components of this new harmonic radar and its first application, the tracking of *Vespa velutina* (yellow-legged Asian hornet). This is an invasive species which, although indigenous to South-East Asia, is spreading quickly to other regions of the world. Because of its fast diffusion and the serious threat it poses to both honeybee colonies and to humans, control measures are mandatory. When equipped with a small passive transponder, this radar system can track the flight trajectory of insects and locate nests to be destroyed. This tool has potential not only for monitoring *V. velutina* but also for tracking other larger insects and small size vertebrates.

## Introduction

Harmonic radars are effective in tracking insects flying at low altitudes and over flat terrain. They have been used in many applications so far^1–3^, from bee neuroethology^4–6^ to classical pollinator ecology^7,8^, from odor-mediated anemotaxis^9,10^ to short range dispersal analysis^11–14^. However, it was found that some were limited in distance^11,13,15–17^, while others worked well only in flat environments^7,18,19^. Handheld systems based on a RECCO transceiver^13,15,16^ are usually included in the harmonic radar category even though they are not able to determine the target range but only measure the received signal strength. We required a way of following an insect in a woody and hilly environment for at least a few hundred metres; to the best of our knowledge, no available harmonic radar could satisfy these needs. This article reports the development and the use of a scanning harmonic radar able to track the flying trajectories of *V. velutina* specimens: the insects are first captured, then equipped with a transponder, and finally released. The goal of this activity is to track *V. velutina* hornets back to their nests in a complex terrain.

The yellow-legged hornet (*V. velutina*) is a social insect native to tropical and subtropical areas of South East Asia^20–22^. Since 2004, the species has spread to other areas of the world such as France^23^, South Korea^24^, and Japan^25^. From France the yellow-legged hornet has spread rapidly to neighbouring countries^26–31^ and is now well established in France, Spain, Portugal, and Italy. It has also been seen in Belgium, Netherlands, Germany, Great Britain, and Switzerland. Forecast models based on climatic data show that many other countries of the world are at risk of invasion from the *V. velutina*^32,33^. This hornet, which is a top predator among arthropods, is particularly harmful because it preys upon honeybees and other native pollinating insects and can lead to the destruction of honeybee colonies^34–37^. Since reaching Europe, *V. velutina* has been wreaking havoc among local apiaries by hovering in front of hives and devouring their occupants. Unfortunately, European honeybees have no natural defences from this opportunistic hunter, it having evolved on the opposite side of the planet. The yellow-legged hornet can spread over large areas and causes so much damage^37–40^ that it has recently been included in the European invasive alien species list (EU Reg. 1141/2016, https://eur-lex.europa.eu/legal-content/EN/TXT/?qid=1545227896749&uri=CELEX: 32016R1141). EU Member States are obliged to adopt surveillance action plans and control strategies against this species (EU Reg. 1143/2014, https://eur-lex.europa.eu/legal-content/EN/TXT/?uri=uriserv:OJ.L_.2014.317.01.0035.01.ENG&toc=OJ:L: 2014:317:TOC).

Since its introduction into France, control of *V. velutina* has been carried out by beekeepers using traps baited with either protein or sugary substances to capture hornet workers^36,41^. Early detection of nests is one of the best available strategies to control the proliferation of *V. velutina*^34^ and to limit its considerable economical damage^42^. However, detection based on visual observation is quite difficult and time consuming^43,44^ since the nests of the yellow-legged hornet are mostly built in hidden places, especially on tree tops and covered by leaves. Moreover, colonies are often spotted late in the year when the reproductive phase is already concluded^45^. For these reasons, a way to discover colonies before they reach the reproductive phase would be of great importance for pest control.

Our innovative harmonic radar can locate nests so they can quickly be destroyed and such a prompt response could eventually halt the spread of hornets as mandated by EU regulations on invasive alien species. A different and successful approach is the radio telemetry system^46^, the main advantages of which are the cost of the system and its portability. However, the tagging procedure is not trivial because of the weight of the active transponder (at least 220 mg). Radio telemetry requires an operator constantly orientating the receiving antenna to follow the radio signal emitted by the tag. There is no accurate feedback about the distance of the hornet and tracking relies on the operator directly following the animal being tracked, which may not be simple in complex terrain.

Our entomological tracking radar could also be applied to study several other insects (honeybees, caterpillar moths, beetles, carabids, butterflies, stinkbugs, etc.) for the broader range of applications mentioned at the beginning of this section. In this respect, radio telemetry has a severe limitation due to the weight of the active transponder.

Our first entomological radar prototype was developed and tested from 2014 to 2016^47,48^. This preliminary system was based on a commercial off-the-shelf transmitting (TX) maritime radar and on our design for a receiving (RX) module. As the TX module was not specifically built for insect tracking, the overall system had a number of limitations, for instance the impossibility of controlling the TX signal. The operating principle of a harmonic radar is to use a passive lightweight transponder to double the fundamental frequency of the signal transmitted from the radar and use it as the receiving signal. Transponders can be detected and located without interference from environmental reflection (clutter) because these unwanted signals are included in the fundamental frequency and can be filtered out. Measuring the time delays between the transmitted and received signals allows one to determine the distances of the transponders from the radar; the direction of arrival corresponds to the pointing direction of the mechanically rotating high directivity antennas in the horizontal plane.

Dealing with the threat posed by *V. velutina* has so far remained challenging as it typically builds its nests up to 20 meters from the ground, concealed in tall leafy trees. However, our innovative harmonic radar offers a new way to spot these well-hidden nests. The main challenge is to obtain reliable detection at long distance (about 400 m) with antennas covering a large field of view (i.e. with low directivity) in the vertical plane. The large field of view in the vertical plane is crucial to be able to detect the transponders both at low and relatively high flight altitudes. Furthermore, this characteristic is vital to operate the radar in hilly and woody areas. Due to these needs and to the low conversion rate of the passive transponders, either the output power of the radar or its sensitivity must be increased to improve the detection range. The frequency of the transmitted signal is a trade-off between the transponder size and antenna horizontal dimension and loss in signal strength (free space path loss). The transponders must be small because they must be attached to an insect’s back without influencing its flying capacities. At the same time, a high directivity antenna (i.e. big in terms of wavelengths) is required to achieve a high resolution in the horizontal plane. On the other hand, free space path loss increases with frequency.

All these requirements drove the development of our innovative harmonic radar, specifically designed in all its parts to track insects. The new TX module allows full control of the way in which the transmitted signals are generated and modulated.

## Results

### System architecture overview

Radar systems generally consist of a TX module and a RX one operating coherently. Our TX module generates a pulse at 9.4 GHz (X-band), constituted by a sequence of modulated sub-pulses; it is then amplified and transmitted via a specialized TX antenna. The antenna has a very narrow beam in azimuth, namely 1.5° Half Power Beam Width (HPBW), and a broader one in elevation, i.e. 24° HPBW. The RX module includes a similar antenna in terms of beam width, but working at the second harmonic, i.e. 18.8 GHz. The received signal is filtered, demodulated and eventually analysed by a Field Programmable Gate Array (FPGA) processing board. The system stores all the transponders’ positions for further replay and geo-localization. A user-friendly graphical interface gives access to most of the radar input parameters (for instance the number of revolutions per minute) and allows to visualize in real time the insects’ movements. Table 1 summarizes the main design parameters, while all details are discussed in section Methods.

**Table 1:** Transmitted signal characteristics.

Peak output power	1 kW
Transmitted frequency	9.4 GHz
Pulse time duration	45 us
Number of sub-pulses	1024
Pulse repetition interval	1 ms
Range resolution	7 m
Revolutions per minute	20

**Figure 1:**
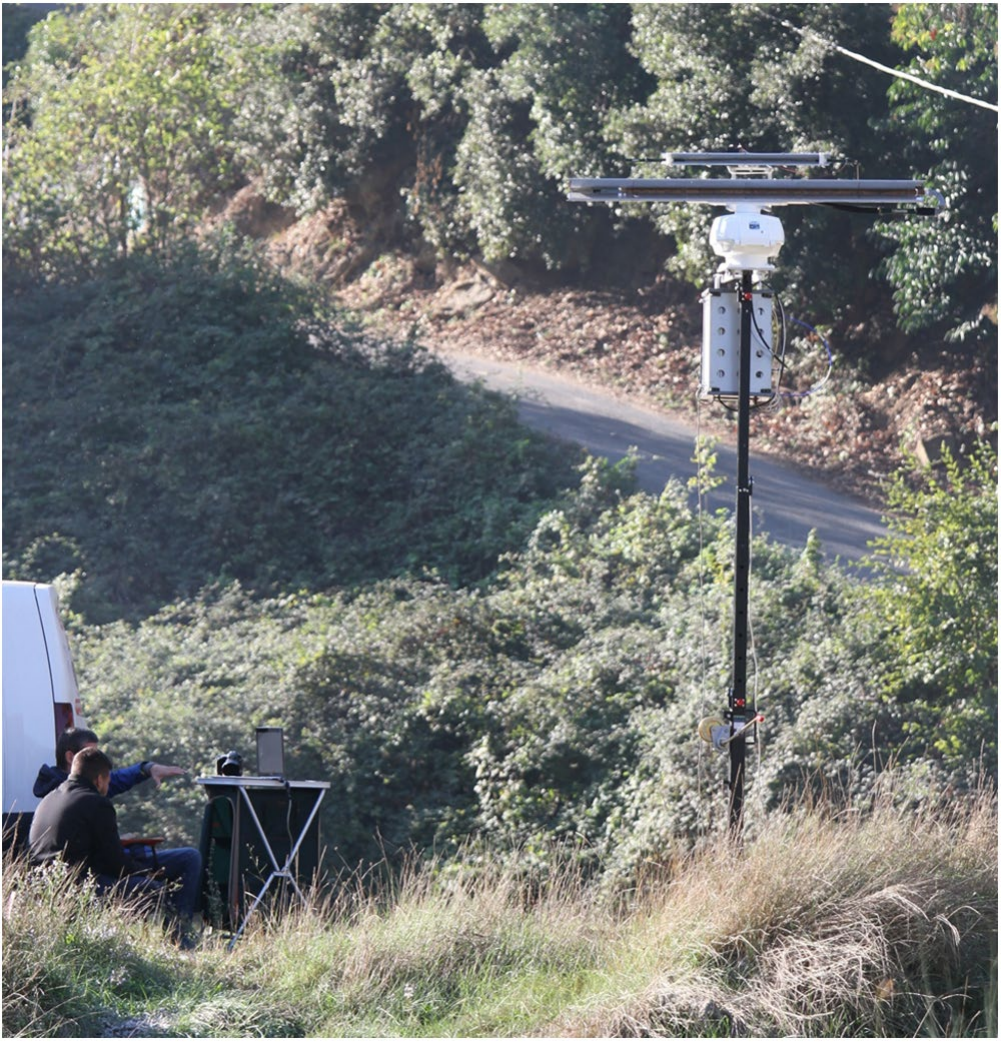
Standard radar setup operating in a hilly and woody environment. The TX and RX antennas are visible on top of the system, while the 4U case rack is hanging right below the rotary joint. A telescopic tower helps to lift the radar up to about 6 metres to avoid obstacles.

The radar hardware is hosted in a 4U rack case, which is mounted on top of a telescopic tower with the mechanically rotating antennas. Figure 1 shows the usual radar setup in operative conditions.

### Transponders

The transponder to be attached to the hornet must be small and lightweight to minimize the effect on the hornet’s movement capability; it must be installed vertically so that it can always be detected by the radar when the hornet is flying. Several types of transponders were tested. The optimized solution is made of a 0.25 mm diameter metallic wire, bent into a “J” shape, with a length of 4 mm and 12 mm for the two arms respectively; a Schottky diode is soldered across the bend. The weight of the transponder is 15 mg, while the overall height corresponds to the longer arm, i.e. 12 mm; the paper pedestal is a 3 mm edge square. Such a light weight can be handled by several insects, from honeybees to bumble bees.

**Figure 2:**
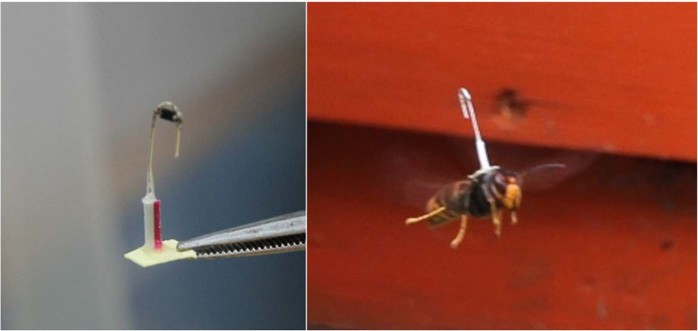
Transponder attached on an Asian hornet’s thorax. The transponder (left) is glued to the hornet’s thorax (right) with the help of a small paper support; an orthodontic glue is required to quickly fasten the tag.

An orthodontic glue was used to attach the transponder onto the hornet’s thorax; this glue polymerizes in few seconds using a high-intensity ultraviolet light lamp. This tool allowed to reliably install the transponder on captured hornets in less than 20 seconds, without any sort of anaesthesia. The hornet workers were captured in front of the beehives with an entomological net, put in a falcon tube, immobilized with a cotton swab and a pair of tweezers, loaded with the transponder and released. This procedure lasted on average less than one minute and it had no impact on the hornet, which started to fly immediately after its release. We also noticed tagged hornets preying in front of the hives up to two weeks after their capture. Figure 2 reports the transponder design (left) and its installation on the Asian hornet (right).

### Field tests

**Figure 3:**
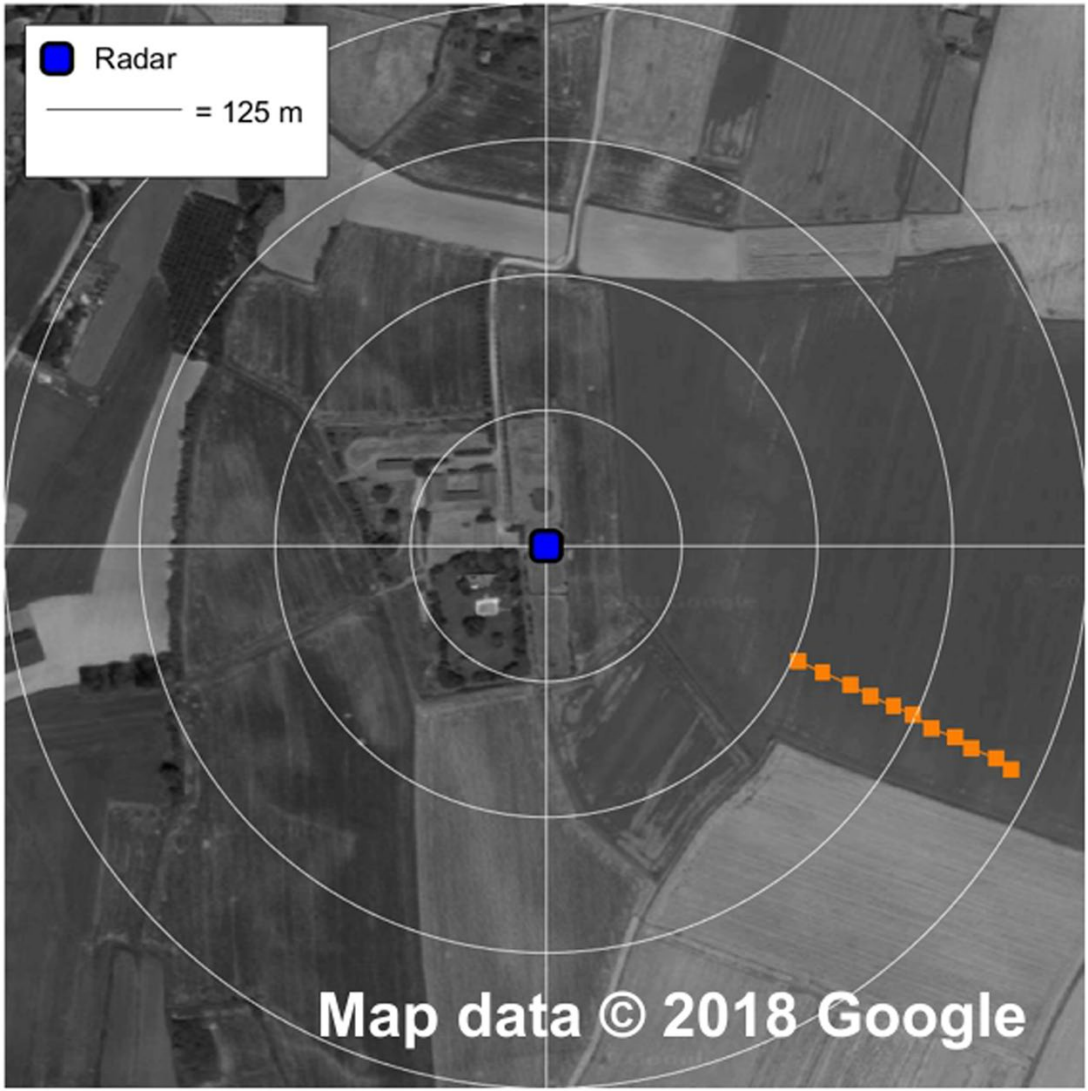
Georeferenced track of the maximum detection field test. The satellite image is taken from Google maps (www.google.it/maps, map data: Google) and edited with Gimp 2.8.20 (www.gimp.org); the measured data and the distance circles are then added with Matlab R2017b (www.mathworks.com/products/matlab.html). The entomological radar is located in the centre of the picture and it is indicated by a blue square; a single track (orange squares) is recorded on flat terrain to find the maximum range of the entomological radar, which is equal to 470 metres in this test.

We first tested our harmonic radar on flat terrain with the goal of checking all the system features, in particular the maximum distance of reliable detection. Figure 3 shows one example of a clear detection at 470 m from the radar position. We routinely and extensively tested our radar in the same conditions to verify the effect of each modification of the system during the design phase.

**Figure 4:**
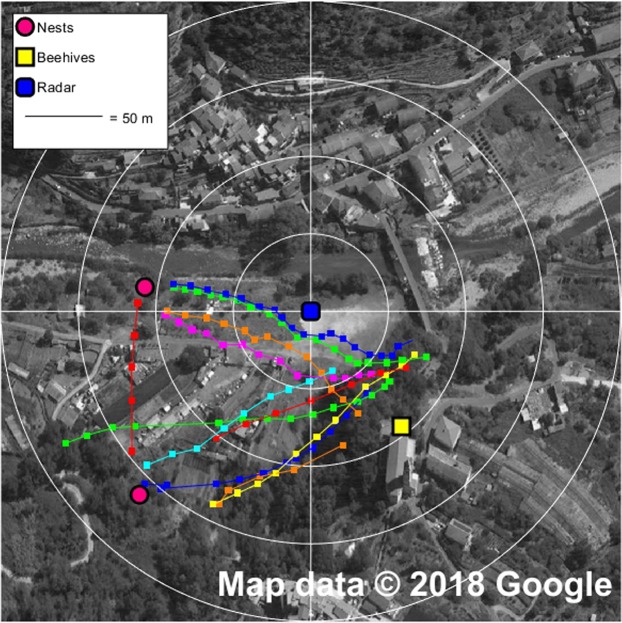
Georeferenced tracks of the Asian hornet near Calvo, in the inland of Liguria in Italy. The satellite image is taken from Google maps (www.google.it/maps, map data: Google) and edited with Gimp 2.8.20 (www.gimp.org); the measured data and the distance circles are then added with Matlab R2017b (www.mathworks.com/products/matlab.html). The entomological radar is located in the centre of the picture and it is indicated by a blue square; beehives and hornet’s nests are marked out with yellow square and pink circles respectively. The small squares along the tracks indicate the exact position of the hornets’ detection. Consecutive concentric circles are located at a reciprocal distance of 50 metres and can be used to evaluate the approximate distance of all detected signals with respect to the radar itself.

Operative field tests took place in September 2017 near Calvo, where the spread of the *V. velutina* is nowadays quite extensive^30^. Despite the difficult terrain, the detected tracks pointed out quite clearly the presence of three arrival and departure spots, corresponding to the known position of the beehive and the unknown positions of two nests hidden in the foliage of tall trees. Most of the tracks reported hornets flying from the nests to the hunting area at a speed of approximately 5 m/s, with a maximum of 6.9 m/s detected once. Figure 4 summarizes the longer recorded tracks georeferenced on the map of the area.

**Figure 5:**
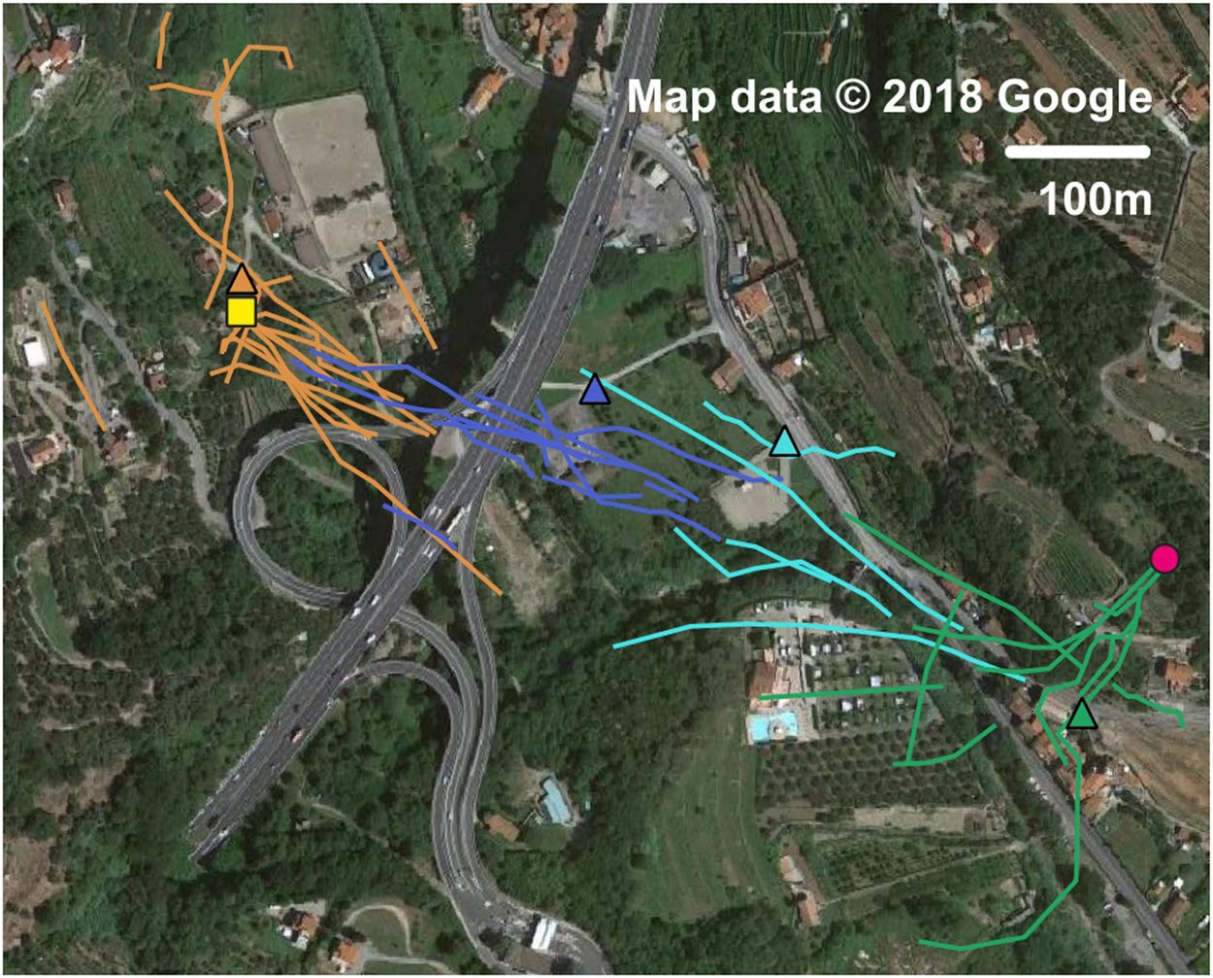
Georeferenced tracks of the Asian hornet near Finale Ligure, in the inland of Liguria in Italy. The satellite image is taken from Google maps (www.google.it/maps, map data: Google) and loaded with QGIS (www.qgis.org/en/site). The positions of the entomological radar are indicated by coloured triangles on the map; the hornets’ tracks recorded for every position are characterized by the same colour. The beehive and the hornets’ nest are marked out with a yellow square and a pink circle respectively. This picture is under the terms of the GNU Free Documentation License, Version 1.3.

**Figure 6:**
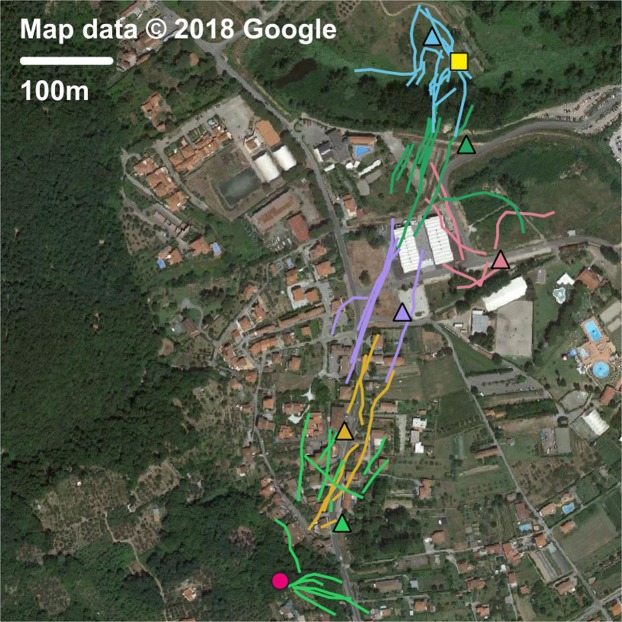
Georeferenced tracks of the Asian hornet near Ameglia, in the inland of Western Liguria region in Italy. The satellite image is taken from Google maps (www.google.it/maps, map data: Google) and loaded with QGIS (www.qgis.org/en/site). The positions of the entomological are indicated by coloured triangles on the map; the hornets’ tracks recorded for every position are characterized by the same colour. The beehive and the hornets’ nest are marked out with a yellow square and a pink circle respectively. This picture is under the terms of the GNU Free Documentation License, Version 1.3.

The 2017 campaign was focused on testing the radar modules in order to guarantee the reliability of all its components from the engineering point of view. Starting from 2018, the radar was routinely used to monitor the flight paths of *V. velutina* and to locate their nests. Systematic recording of traces was carried out and a database was stored for future analysis, including all the operative parameters such as the exact number of hornets tagged per session; a paper dedicated to the entomological analysis of the 2018 operations will be released soon. Figures 5 and 6 report the traces recorded in Finale Ligure and Ameglia, as an example of the radar effectiveness in different environments. In both cases our harmonic radar led to the localization and destruction of a nest, 561 m from a beehive in Finale, 786 m from a beehive in Ameglia.

## Discussion

A new innovative harmonic radar has been developed and tested; this prototype is capable of tracking the flight trajectory of insects, equipped with a small and lightweight passive transponder, in a hilly and woody environment. All the parts of this prototype have been designed and manufactured or procured. The final system consists of a 4U rack case, containing most of the hardware, connected to the rotating antennas and operated on a trellis; a graphical user interface running on a laptop PC manages all radar outputs and input parameters.

The tests on flat terrain demonstrated an effective 470 m maximum range of operation. The first nests of *V. velutina* were identified in late 2017 by following the georeferenced hornets’ tracks. During 2018, our harmonic radar was extensively used in the field campaigns; two examples are reported in this paper, documenting its effectiveness in different environments. All data related to 2018 operations were stored in a database that will be analysed from the entomological point of view in a future paper. The usability and the flexibility of the system are satisfactory, as demonstrated by daily operations; the current version requires approximately 20 minutes to be installed (and uninstalled) by a single operator, 15 minutes by two operators. The radar does not need the constant presence of an operator when active, allowing him/her to capture and tag the insects instead. One hour is usually enough to have a clear indication on where to move the radar next in order to get closer to the target (the hornets’ nests in our validation example). This time can be reduced to 30 minutes or even less in case of more than ten tagged insects. The nest can be located with the 7 m resolution of the radar in the most favourable case, i.e. when it is directly visible from the radar position. Our harmonic radar can be used to narrow the search area if the nest is well hidden within a forest.

The main limitation of this prototype is the need for a vehicle to move the radar equipment from one location to the next one; the overall weight (the radar and the trellis) is about 50 kg, which prevents its positioning too far from the vehicle and hence from a road or a trail. A few upgrades are foreseen in this respect, i.e. the possibility to use the radar directly from a motorized vehicle and the design of a lighter portable unit. The system engineering for the production of more units is being considered as well.

This radar system is presently adopted as one of the measures to eradicate *V. velutina* from Italy, but it can be adopted as part of an early-warning strategy valuable in countries where this hornet can still be stopped before it gains a foothold. Our harmonic radar can identify the home range of *V. velutina* and provide important information on the biology of this insect; such information can also be useful to improve the control of the Asian hornet. The extent of the damage created by *V. velutina* remains under study, but initial results suggest that 30% of bee colonies attacked face complete collapse^36^. Mankind depends on millions of honeybee colonies to produce honey and pollinate crops^49^, which must be preserved from this threat.

The radar usage can be extended to many other applications involving the study of the biology of other insects and animals of limited size, such as bees, bumblebees, butterflies, dragonflies, moths, etc. The only requirement is the capability of carrying a small and lightweight transponder (about 15 mg of weight).

## Methods

### System architecture: the harmonic radar

A harmonic radar is a system that illuminates a region of space with radiofrequency waves and receives the harmonics of the transmitted frequencies generated by any non-linear device present in the environment. The second harmonic is usually the strongest received one. The received signal is processed to find the exact locations (range and direction) of the points causing the generation of these harmonics. The harmonic radar technique is necessary to be able to detect the targets and to suppress all the possible clutter from the ground features, vegetation, etc. Entomological radars exploit these characteristics by installing a small lightweight passive transponder constituted by a wire and a diode (non-linear device) on the insects to be tracked.

High range resolution and high sensitivity, as may be obtained with a transmitted short pulse, are important for all radar applications, in particular for harmonic radars where passive transponders have very low efficiency. The main limitation in achieving high sensitivity with short duration pulses is that a high peak power is required for a large pulse energy. We use a transmitted long pulse to solve this problem; the long pulse is modulated in frequency or phase and the received echoes are processed with a proper matched filter without losing the short pulse advantages. This is called pulse compression.

A long pulse of duration T is divided into N sub-pulses, each of width τ; an increase in bandwidth is achieved by changing the phase of each sub-pulse. A common form of phase change is binary phase coding, in which the phase of each sub-pulse varies between two different values according to some specified criterion. The pulse compression ratio equals the number of sub-pulses N = T / τ = BT, where the bandwidth B is approximately equal to l/τ. The filter output is a compressed pulse of width τ with a peak value that is N times the one of the uncompressed pulse. In this case, the optimum filter is implemented as the correlation between the received echoes and the transmitted sequence. An advanced Doppler filtering technique is also implemented to further suppress the local leakage and the remaining clutter.

**Figure 7:**
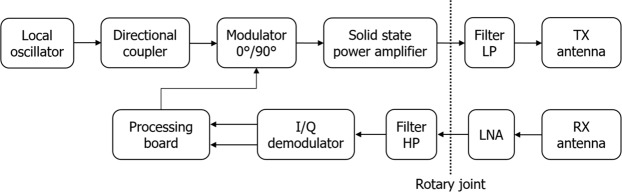
Harmonic radar block diagram. The rotary joint dashed vertical line divides the rack case containing most of the electronics from the rotating head of the radar, which is made up of the Low Pass filter, the transmitting and receiving antennas, and the Low Noise Amplifier.

Our harmonic radar system is made up of a transmitting (TX) module and a receiving (RX) module operating coherently or, otherwise said, sharing the same local oscillator. Figure 7 reports the overall block diagram of our entomological radar.

### System architecture: the TX module

The TX module of our harmonic radar includes a 9.4 GHz dielectric resonator local oscillator. Dielectric Resonator Oscillators (DROs) are microwave oscillators that use a dielectric resonator as frequency stabilizing element in order to achieve excellent frequency stability, very low phase noise and very good second harmonic suppression. We chose the Raditek R-DRO-B model. We also needed a directional coupler, connected to the DRO, to divide the power between the TX modulator and the RX demodulator according to a defined ratio. We selected the NARDA 4247B10 10 dB directional coupler that presents a very limited insertion loss.

The core of the TX module is the Binary Phase Shift Keying (BPSK) modulator. Since the transponder doubles both the frequency and the phase of the incoming signal, the modulator has been specifically designed for this harmonic radar with the two phases equal to 0° and 90° (instead of the classical 0° and 180°). The modulator is based on two Hittite HMC347 switches, the first one implementing the on/off keying and the second one taking care of the choice of the output signal between two branches with quarter wavelength difference. The device includes the drivers for the two switches, which receive the commands from the FPGA processing board. The modulated signal is then amplified to 1 kW by a Gallium Nitride (GaN) Solid State Power Amplifier (SSPA) produced by COMTECH PST (BPMC928109-1000).

The amplified signal is sent to the transmitting antenna through a dual channel rotary joint. The 9.4 GHz TX antenna is a 150 cm long slotted WR90 waveguide with 50 radiating elements (horizontal slots, vertical polarization) tapered along the waveguide direction and entirely designed and manufactured in our laboratory. The antenna mounts two flanges in order to implement a vertical sectoral horn and, therefore, to get a more accurate radiation pattern in the vertical plane.

### System architecture: the RX module

The RX antenna collects the returned signals from all transponders, doubled in frequency and phase. The 18.8 GHz RX antenna is a 100 cm long slotted WR51 waveguide with 50 radiating elements (horizontal slots, vertical polarization) tapered along the waveguide direction and entirely designed and manufactured in our laboratory. As for the TX launcher, also the RX antenna mounts two flanges to have a more accurate radiation pattern in the vertical plane.

A Low Noise Amplifier (LNA) is directly connected to the RX antenna; it has high gain and it is manufactured by BZ Technologies. Since the LNA is integrated with the rotating RX antenna, a dedicated battery is required for power. The signal is then passed through the dual channel rotary joint and sent to a BPSK coherent I/Q demodulator.

The I/Q demodulator is the Hittite HMC570, which hosts an active x2 multiplier that can be directly connected to the TX DRO. The demodulated I (In-phase) and Q (Quadrature phase) signals are digitized and delivered to the FPGA processing board.

The Brush Less DC (BLDC) electric motor, responsible of the mechanical rotation, is controlled by the same FPGA processing board through a motor control unit. The rotational speed of the antennas is usually set to 20 rotations per minute.

### System architecture: advanced radar analysis

The FPGA processing board hosts the developed firmware that correlates the received signals with the transmitted sequence in real time, 1000 times per second. The results of the correlation process are sent through an Ethernet connection to a standard laptop PC for the high level processing and output visualization. On the PC, the data are organized in a bi-dimensional matrix per each transmitted pulse, where the I and Q signal levels are stored per each range bin (of about 1 m) and angle of arrival bin (of about 0.1°).

The core of the high level processing is the Doppler shift estimation; it is implemented by performing a Fast Fourier Transform (FFT) per each range-angle bin along a certain programmable number of pulses (usually set to 32). The total number of bins to be analysed is equal to 500 (range) × 3000 (angle) × 32 (Doppler) = 48 million bins per each antennas’ revolution!

The output of the FFT is in the Doppler frequency domain, in which the separation into a certain number of bands offers a very flexible approach to further discriminate against fixed and moving targets. Moreover, an adaptive threshold after the FFT may be set if moving clutter (such as that from weather, birds or waiving vegetation) appears with a non-zero mean Doppler shift. A maximum detection in the Doppler frequency domain is performed after thresholding; the magnitude of the maximum peak is averaged and plotted on a plan position indicator.

### Field tests

During the design phase of our harmonic radar, systematic testing on flat terrain was performed to check the impact of each design upgrade. In those tests the transponder was glued vertically on the top of a 2 m long wooden stick (in the same way as for the hornets’ thorax) and handled by a person walking along a specific path, as documented in Fig. 3.

The first operative on-field test took place in 2017 in Calvo, which is located in the inland of Liguria region in Italy, not far from the French border. The area under analysis was about 300 m by 300 m, presented some slopes of up to 20% gradient and had a moderate amount of tall trees and other obstacles (poles, fences, houses, etc.). We captured 47 hornets in 58 minutes starting from 11:30 am; we glued the transponders on their thoraxes, then released them and observed their movements in real time, recording more than 100 tracks. Figure 4 summarizes the longer recorded tracks georeferenced on the map of the area.

Figure 5 reports the recorded traces from Finale Ligure, located again in Liguria region, about 100 km west of Calvo. The entomological radar was operated in early October 2018, in a mostly rural area where the Asian hornet is quickly spreading. The radar was initially placed in view of a beehive (yellow square), then it was moved three times during three consecutive days (coloured triangles) where the tracks faded due to the orography. 21 hornets were tagged at the beehive location, 14 the first day, 7 the last one. The radar was operated for approximately 10.5 hours in total.

The second 2018 example refers to an urban area, close to Ameglia, in the western part of Liguria. In that area *V. velutina* is almost absent (only two nests have been reported in 2018). The traces were recorded in mid September, during a two day on-field campaign; 17 hornets were tagged during the first day, 4 during the second. The radar was initially positioned close to a beehive and moved three times during the first day, as documented in Fig. 6. On the morning of the second day, the radar was first operated from the last position, then moved again two times. A nest was detected before noon, at about 786 m from the beehive, after 12 hours of radar operations.

## Data Availability

The data analysed in this paper are available from the corresponding author upon request.
